# Conflict and Emerging Infectious Diseases

**DOI:** 10.3201/eid1406.080027

**Published:** 2008-06

**Authors:** Louise A. Kelly-Hope

**Affiliations:** * Liverpool School of Tropical Medicine, Liverpool, UK

**Keywords:** conflict, cholera, outbreak, letter

## To the Editor

In the November 2007 issue of Emerging Infectious Diseases, Gayer et al. (*1*) describe how conflict leaves populations in dire poverty, internally displaced or seeking asylum, having poor access to essential services, and consequentially vulnerable to infectious diseases.

Cholera, caused by the bacterium *Vibrio cholerae,* is a disease that seems particularly sensitive to conflict and deserves more consideration. Major risk factors for cholera—poverty, overcrowding, poor hygiene, contaminated food, and lack of safe drinking water (*2*,*3*)—largely resemble the consequences of war and civil fighting. Yet little is known about the relationship between cholera and conflict. This lack of information may be because cholera tends to be epidemic, affecting hundreds to thousands of people across vast, war-torn regions, making it impossible for local governments, nongovernment organizations, and aid workers to control, let alone collect and analyze data.

Examination of data sources listed by Gayer et al. (*1*) and recent reviews (*2*,*3*) indicate that cholera occurs 1) in countries during war and civil unrest, as exemplified by the latest outbreaks among displaced populations across northern Iraq; 2) in neighboring countries, where temporary camps accommodate masses of political refugees under poor conditions, such as those in eastern Chad near Darfur, Sudan; and 3) during the postwar period when large numbers of repatriated persons return home and consequently place undue pressure on an eroded and fragile national infrastructure, as evident in Angola in recent years.

Moreover, all the countries affected by conflict shown in the Appendix Figure by Gayer et al. (*1*) have reported cholera outbreaks (*2*–*4*). They are also among the poorest countries in the world; the latest statistics on human development (*5*) indicate that compared with all developing countries, on average they have higher rates of undernourishment, refugees, child deaths, and less adequate water and sanitation facilities. Thus, more information is needed about conflict and cholera, especially in Africa.

## Supplementary Material

Appendix Figure Geographic distribution of recent emerging or reemerging infectious disease outbreaks and countries affected by conflict, 1990–2006. Countries in yellow were affected by conflict during this period (source: Office for the Coordination of Humanitarian Affairs, World Health Organization, www.reliefweb.int/ocha_ol/onlinehp.html). Symbols indicate outbreaks of emerging or reemerging infectious diseases during this period (source: Epidemic and Pandemic Alert and Response, World Health Organization, www.who.int/csr/en). Circles indicate diseases of viral origin, stars indicate diseases of bacterial origin, and triangles indicate diseases of parasitic origin. CCHF, Crimean-Congo hemorrhagic fever; SARS-CoV, severe acute respiratory syndrome coronavirus
