# Supplementary figures and images for: Conflict and Emerging Infectious Diseases
Source: Emerg Infect Dis. 2008 Jun;14(6):1004–5. doi: 10.3201/eid1406.080027 (PMC2600301; doi:10.3201/eid1406.080027)

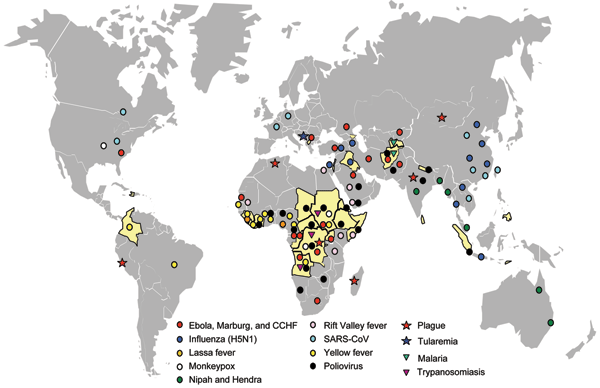

Supplement: Appendix Figure — Geographic distribution of recent emerging or reemerging infectious disease outbreaks and countries affected by conflict, 1990–2006. Countries in yellow were affected by conflict during this period (source: Office for the Coordination of Humanitarian Affairs, World Health Organization, www.reliefweb.int/ocha_ol/onlinehp.html). Symbols indicate outbreaks of emerging or reemerging infectious diseases during this period (source: Epidemic and Pandemic Alert and Response, World Health Organization, www.who.int/csr/en). Circles indicate diseases of viral origin, stars indicate diseases of bacterial origin, and triangles indicate diseases of parasitic origin. CCHF, Crimean-Congo hemorrhagic fever; SARS-CoV, severe acute respiratory syndrome coronavirus [file 08-0027_app-s1.gif]
