# Supplementary material for: Conflict and Emerging Infectious Diseases
Source: Emerg Infect Dis. 2008 Jun;14(6):1004–5. doi: 10.3201/eid1406.080027 (PMC2600301; doi:10.3201/eid1406.080027)
Supplement: Related texts

# Reply: In Response

**Authors:** Michelle Gayer, Dominique Legros

**Affiliations:** * World Health Organization, Geneva, Switzerland

We agree with Kelly-Hope on the propensity for cholera outbreaks to occur in conflict-affected countries and the need to monitor and respond more effectively to such events. In 2006, cholera was reported from 33 countries in Africa, and 88% of all reported cases were from conflict-affected countries (*1*).

As highlighted in our November 2007 article on conflict and emerging infectious diseases (*2*), conflict situations present a multitude of risk factors that enhance disease emergence and transmission, over and above those in other resource-poor countries. Many such conflicts facilitate the occurrence of cholera outbreaks.

More precise research on cholera and conflict is indeed necessary. However, despite cholera being a disease that has been around for a long time and that causes frequent outbreaks to this day, much information about this disease, beyond its relationship with conflict, remains unknown. For example, although *Vibrio* *cholerae* persists in the environment, little is known about the exact conditions that trigger a cholera outbreak at a particular time. Further elucidation is needed about the factors that influence the duration of an outbreak, disease severity, and duration of individual protective immunity after an episode of cholera.

Cholera, which is closely linked to a country’s social and economic development (*1*,*3*), ceased to be of concern in Europe, for example, when access to potable water and sanitation improved although its cause was still unknown and antimicrobial drugs were not yet available. Today, renewed interest from the international public health community is urgently warranted, and strong initiatives are needed to help developing countries (conflict-affected or not) fight against cholera and control this easily preventable disease on a global level.

## References

[1] World Health Organization. Cholera 2006. Wkly Epidemiol Rec. 2007;82:273–84.17679181

[2] Gayer M, Legros D, Formenty P, Connolly MA. Conflict and emerging infectious diseases. Emerg Infect Dis. 2007;13:1625–31.1821754310.3201/eid1311.061093PMC3375795

[3] Anbarci M, Escaleras M, Register C. From cholera outbreaks to pandemics: the role of poverty and inequality. Working Papers series no. 05003. Boca Raton (FL): Florida Atlantic University; 2006 [cited 2008 Feb 29]. Available from http://home.fau.edu/mescaler/web/working%20papers/Cholera.pdf
